# The Promise of Integrating Health and Corrections Datasets: A Short Report on Familial Impact of Parental Justice System Involvement

**DOI:** 10.1007/s12103-026-09902-6

**Published:** 2026-03-31

**Authors:** Aaron Murnan, Henry Duah, Nichole Nidey, Julie Wijesooriya, West End Community Research Advisory Board (WE-CRAB), Sarah Manchak, Samantha Boch

**Affiliations:** 1 Martha S. Pitzer Center for Women, Children and Youth, The Ohio State University, 1577 Neil Avenue, Columbus, OH 43210 USA; 2 College of Nursing, Marquette University, 1225 W. Wisconsin Avenue, Milwaukee, WI 53233 USA; 3 Department of Epidemiology, College of Public Health, University of Iowa, 145 N. Riverside Drive, Iowa City, Iowa, 52242 USA; 4 Center for Clinical and Translational Science and Training, University of Cincinnati, 240 Albert Sabin Way, Cincinnati, OH 45229 USA; 5 School of Criminal Justice, University of Cincinnati College of Education, Criminal Justice, and Human Services (CECH), PO Box 210389, Cincinnati, OH 45221 USA; 6 Center of Nursing Excellence and Center of Child Health Equity and Outcomes Research, Abigail Wexner Research Institute, Nationwide Children’s Hospital, Columbus, OH 43205 USA; 7 College of Nursing, University of Cincinnati, Cincinnati, OH 45221 USA

**Keywords:** Criminal justice involvement, Intergenerational effects, Health and justice, Methodologies, Big data

## Abstract

This case study describes the development of a novel cross-sector administrative data linkage in a large Midwestern city, which connected maternal justice involvement records from state and county systems with mother–child health data from adult and pediatric healthcare institutions. Designed to overcome barriers in studying intergenerational impacts of criminal justice involvement, the project was conducted in two phases: creation of a maternal justice dataset using public records; and linkage to a regional perinatal health repository via an honest broker. The resulting de-identified dataset enables research on mechanisms by which maternal justice involvement affects child health and healthcare use. Guided by a community research advisory board and approved by **IRB masked**, the study emphasized ethical considerations and minimizing potential harm. Despite limitations, this work offers replicable, cost-effective models for integrated data use that can contribute to national efforts to improve outcomes for justice-involved families through cross-sector collaboration and data-informed policy innovation.

## Introduction

National organizations have increasingly emphasized the importance of leveraging individual indicators of health and well-being within corrections research and practices, most recently within calls to re-envision the long-standing emphasis on recidivism as the mark of individual and program success (The Council of State Governments Justice Center, 2025). Tracking and reporting a broader set of holistic health measures requires significant investment, coordination, and collaboration across community, carceral, state, and health agencies. Since 1985, only 0.12% of all projects funded by the National Institutes of Health have addressed “incarceration” and 0.15% have addressed “re-entry and recidivism” (Boch et al., 2023). This dearth of funding has undoubtedly contributed to a lack of best health practices and guidance for youth and families affected by justice-system involvement. Beyond a lack of federal investment, health and criminal justice professions have been historically siloed, despite their shared aim and prioritization of rehabilitation. A lack of communication, collaboration and training across sectors also contribute to the difficulty.

One way to address these persistent challenges would be to link administrative correctional and health records; however, patient health information is rightfully protected by federal law and difficult to access, especially for scholars outside of health-related fields (such as those in criminal justice). Reciprocally, correctional data is difficult to access and maintain for many health researchers. In turn, there are few health-justice administrative data linkages that exist to enhance the capacity of these fields to intersect and study critical topics of interest such as the impact of incarceration on individual, family, and community health. Cross-sector data linkages at the county, state, or national levels would maximize use of existing, reliable data to inform health and the delivery of healthcare services to justice-involved families. Unfortunately, siloed professional fields and the lack of data integration prevails.

These structural-, professional-, and data-level siloes are especially problematic given the size of the United States (US) criminal legal system (CLS). About *37% of all US adults* (or 77 million adults) have records of crime with direct and indirect consequences on family and community health. Over half of all incarcerated US adults are parents of children under 18 years (52–63%; Glaze & Maruschak, 2008; Maruschak, 2021), and approximately one in every 14 youths have had a parent incarcerated (Murphey & Cooper, 2015), equating to about two students in a typical classroom. Justice system involvement has substantial health consequences to individuals and their families/children, as it directly impacts an individual’s ability to obtain employment, income, meet basic needs, and maintain safe housing (Anne Milgram, April 2018; Pager, 2003). Even so, very little is known about the health and well-being of children and families for which a parent becomes justice involved.

Research on the effects of parental incarceration has largely relied on national health surveys, which, despite their feasibility advantages, lack critical details on justice involvement that ultimately undermine scientific rigor (e.g., offense type, incarceration frequency, duration, and parenting gender; Wildeman et al., 2018). Surveys are further limited by sampling biases, reliance on self-reporting of stigmatized experiences and circumstances, as well as the exclusion of various children, such as those in out-of-home placements (Campbell et al., 2020; National Academies of Sciences & Medicine, 2019a, 2019b, 2024). It is well known that stigma, distrust, and fear of repercussions contribute to widespread underreporting, especially in health care settings where screening for parental incarceration is rare and provider training is insufficient (Mersky et al., 2019; National Academies of Sciences & Medicine, 2019a, 2019b). Further, self-report data from parents actively involved in the justice system are also likely to suffer from social desirability and lack of knowledge due to fear of consequences or disconnection with family and children due to incarceration. These limitations may obscure associations and bias findings towards the null, which further undermines on-going efforts to understand and quantify the true impact of incarceration on the health and well-being of children and families.

Leading scholars have called for substantial investments in integrated data systems to link objective and reliable data from healthcare records to justice records to better capture exposure and health outcomes, as well as to understand and intervene on potential health effects and mechanisms for which parental incarceration impacts children and families over time (Lee & Wildeman, 2021; Wildeman et al., 2018). These calls align with both broader recommendations from the National Academies for cross-sector collaboration to ensure all children have the opportunity to thrive and recent priority initiatives to re-imagine the measurement of re-entry success and family wellness when a parent becomes involved in the CLS (National Academies of Sciences & Medicine, 2019a, 2019b; The Council of State Governments Justice Center, 2025). Even so, these calls remain largely unanswered in most cities across the country.

Although progress in this direction has been slow, the few case examples have shown promise: one adult health care system in the US has linked hospital claims data to adult arrest data (Camden New Jersey; Anne Milgram, April 2018) and one state (Wisconsin) has developed a repository of linked administrative records (Wisconsin Administrative Data Core), including linked data from child welfare, criminal legal, education, public assistance, housing, etc.; which are updated annually and include records for more than 13 million individuals (Brown et al., 2011) On an international scale, cross-sector data linkages of survey and clinical data to multiple nationwide government administrative databases (including crime) and electronic medical records for children and adults exist in New Zealand (Caspi et al., 2016; Richmond-Rakerd et al., 2020) and in Denmark (Erlangsen & Fedyszyn, 2015). Findings from the Dunedin and New Zealand linkages indicated that a relatively small population segment accounted for high-need, and high-use across both health and criminal justice sectors (Caspi et al., 2016; Richmond-Rakerd et al., 2020). There are numerous policy implications for co-locating segments of the population that experience high use across systems, especially for youth, families, and communities. We also know that there are shared individual and family risk factors in the development of poor health, and the conduct or ongoing involvement of crime or violence (e.g. poverty, food insecurity, poor mental health care access, racism, etc.). Establishing cross-sector data linkage throughout the US that integrates reliable and objective parental and child health data with administrative correctional records can enable novel and rigorous inquiry into the effects and mechanisms of parental justice involvement on child health. Until cross-sector data linkages become wide-scale, innovative approaches to creating health-justice data linkages locally could be explored.

The current case study sought to establish a novel cross-sector administrative data linkage in one large Midwestern city between a) administrative criminal justice data collected by the study state’s Department of Rehabilitation and Corrections and the study county’s court system and b) an existing mother–child dyad health linked dataset housed jointly at an adult health hospital system (e.g. labor and delivery records) system and a pediatric-based hospital institution. We outline the process and methods for establishing this cross-sector data linkage inclusive of methods implemented, challenges encountered, and next steps that can guide others seeking to create novel health justice data linkages in their respective state or county. It is important to note that the design of this proposal, its execution, and use have been iteratively informed by a community research advisory board, which has offered key guidance on ethically establishing the data linkage and minimizing potential unintentional harm. While criminal justice involvement is a national problem, this work is a particular concern in the study state, which ranks 17th in the nation for the percentage of youth with an incarcerated parent (Child and Adolescent Health Measurement Initiative, 2022–2023). Unfortunately, for the past 40 years, the study county has incarcerated more people per capita *every year* compared to the state and national rates (Vera Incarceration Trends, 2024). As such, this project is not only timely and important within a national context, but it has unique significance to our state and local community.

## Methods and Process

### Overview Summary

We sought to establish a cross-sector data linkage between (a) female justice involvement data collected by our state’s Department of Rehabilitation and Corrections and county-level court system; and (b) mother–child dyad health data housed at a large midwestern city’s adult hospital system (e.g. labor and delivery records) and child medical records from a pediatric-based hospital institution within the same county. We conducted the linkage process in two phases and obtained approval by **Masked for Review** Institutional Review Board (Protocol 2023–0124). Ancillary reviews by a pediatric/minor representative and a ‘prisoner’ representative were also included in the IRB review to aid the responsible conduct of this research at this institution, and both were positive in their assessment on the importance and significance of such cross-sector approaches to better investigate how to best support families affected by maternal incarceration. The study was considered “no greater than minimal risk” due to the use of an honest data broker (or third party) to link the datasets and then provide a de-identified dataset for use to the research team.

The first phase was conducted in 2022–2023 (**Phase 1** – *Community Feedback and Creation of a Maternal Justice Records Dataset)*, and the second phase was conducted in 2023–2024 (**Phase II** – *Honest Broker Linkage of the Maternal Justice Infant Data Hub.*). Both phases were funded by internal awards offered by **Masked for Review**. The initial data linkage conceptualization was conducted in partnership with a local community research advisory board that included adults with personal and family-based experiences of incarceration. Upon agreement of creation and use, a graduate student created a dataset of administrative justice records of women with active justice involvement from the county area of interest using publicly available incarceration records. Then, an honest broker linked the maternal justice records collected to the Maternal Infant Data Hub (MIDH) using first and last names, date of birth, racial identity, and gender. MIDH is a dataset that contains maternal and infant electronic medical records from a large adult and pediatric health system in the study county.

### Phase 1 – Community Advisory Board Feedback and Creation of A Maternal Justice Records Dataset

#### Community Research Advisory Board (RAB)

Prior to creating a health-justice linkage, we sought to obtain feedback from community members to identify and address potential community concerns. As such, we met with a local Research Advisory Board (RAB; **name and website reference masked for anonymous review**;) to obtain feedback about our proposed ideas and tentative data linkage of publicly available state and county level criminal justice data with an existing data repository of maternal and child health records housed within a large midwestern medical center. Formed in 2016, the RAB consists of members who are active in their community and have been trained in research ethics and methods. The members of this advisory board have worked with multiple institutions and numerous investigative teams to inform and improve research conducted in and about their communities (Jones et al., 2022; Kues et al., 2022).

Overall, our proposed linkage was well-received, and all RAB members expressed desires to promote health among children of justice-involved parents. We obtained valuable feedback regarding a) perceptions of the idea (e.g. high support); b) concerns on the primary use of the dataset and lack of direct benefits to those whose data is accessed; as well as c) thoughtful ways to expand this research. The RAB emphasized the critical importance of protecting families by ensuring neither physicians nor researchers gain access to identifiable linked data that could impact or identify these families within the context of their pediatric care settings. RAB assisted with the conceptualization of the overall study design, sensemaking, ethics, and approved the summarization of the process and methods.

#### Creation of a Maternal Justice Records Dataset

As part of Phase I, we also created a dataset of all actively justice-involved women (currently detained, on probation, on parole) in the state leveraging publicly available criminal legal data sourced from the (1) **state masked for anonymous review**Department of Rehabilitation and Correction (**DRC**) and (2) **county masked for anonymous review** **Clerk of Courts (CC) publicly available records**. The DRC maintains publicly available data regarding basic demographics, admission date, correctional institution, status, offense(s), and offense information (e.g. jail time, term length, and degree of felony). This data is stored as an interactive list, for which clicking on each individual’s name will populate all aforementioned data points. It is important to note, that DRC records only include those actively justice-involved (actively incarcerated, released on probation or parole, or those who died while incarcerated). A trained graduate student extracted all public DRC data on all females in the dataset with a matching study county of commitment during the summer of 2022. The DRC data was entered into an excel sheet and reviewed for accuracy. The DRC justice records dataset included information on 320 females with active justice involvement with a commitment from the study county.

The graduate student then searched CC records for all prior charge records for each of the individuals located/identified in DRC to provide contextual data on their prior criminal legal involvement within the county. The CC maintains publicly available records for all historical criminal charges and arrests within the county. However, this data is not warehoused or stored in an organized fashion where it can readily be analyzed. We also inquired with CC directly about providing data on all females with arrest records but was told by county administrators that there was no way to retrieve this information despite their public availability. Therefore, all data was queried for specific individuals using first and last name and then verified using date of birth, racial identity, and gender. County records include prior charges, arrests, case numbers, and court dispositions within the county. Approximately 189 of the 320 women had information from the County Criminal Court records, including charges and sentences. Records that contained a lack of complete information, date birth number discrepancies, and discrepancies with misspellings of names by a letter – limited our ability to confirm many records.

### Phase 2 – Honest broker linkage to the Maternal Infant Data Hub

In Phase II, our created criminal justice record dataset was then merged with an existing data repository, the Maternal Infant Data Hub^18^ (MIDH) using “Honest Brokers”.

#### Maternal Infant Data Hub

The Maternal Infant Data Hub (MIDH) was developed in 2014 to establish a perinatal data repository to spur innovative population-based maternal-child research through linking 1) maternal healthcare records from a large institutional adult healthcare facility with 2) pediatric records at a large institutional pediatric healthcare facility within the same county (Hall et al., 2018). Maternal data is linked to the infant data using infant’s name, sex, date of birth, address, birth weight, and parental name(s). The regional perinatal data repository (containing over 60,000 mother-infant dyads) includes integrated notes (physician progress, nursing, and social work notes), diagnostic and procedural codes, medication orders, and state vital record data for mother and child (Hall et al., 2018). Data also includes delivery, postnatal care, and all follow-up health and care data from 2014 to the present and is updated quarterly. Detailed information on the creation (Hall et al., 2014, 2018) and use of the Maternal Infant Data Hub are published elsewhere (Arter et al., 2021; Nidey et al., 2022).

#### Honest brokers – Data Management and Analysis Center (DMAC) at **Masked to Support Blind Review**

**Fig. 1:**
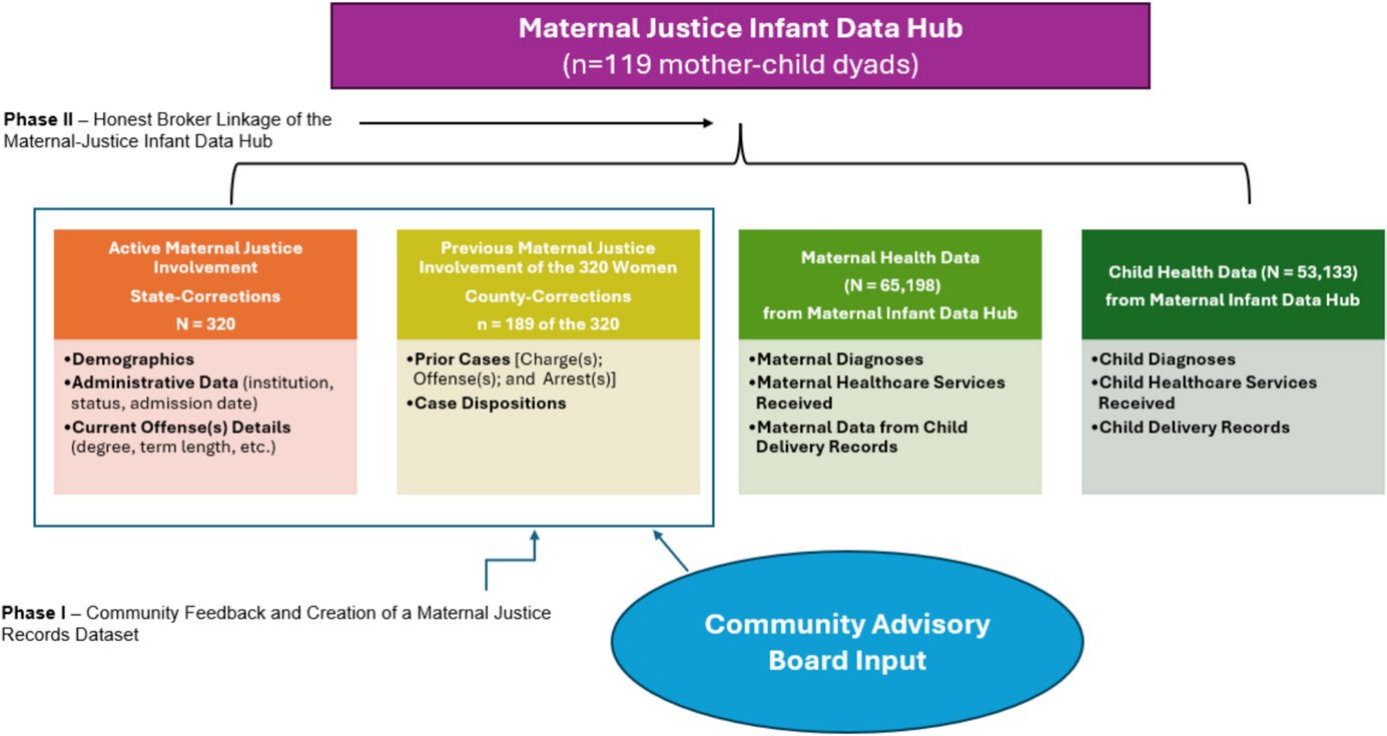
Maternal Justice Infant Data Hub

In line with RAB recommendations, the DMAC served as ‘honest brokers’ of the data merge and established the linkage to limit potential risk to families by preventing the study team or healthcare providers from accessing identified linked data. Specifically, DMAC programmed and merged identifiable data from the MIDH and the identifiable (but publicly available) justice-involvement data created by the study team. The linkage leveraged full name, date of birth, gender, and racial identity of mothers in MIDH and the women in the justice records dataset matched 1:1. The linked dataset was de-identified by DMAC and then made available to the research team to conduct analyses. This was a critical and necessary step to safeguard identifiable information, while establishing this critically needed cross-sector data linkage. The MIDH repository used at the time of the linkage (November 2023) contained information on approximately 65,198 children and 53,133 mothers. The Maternal Justice Infant Data Hub (MIJDH) contained information for approximately 77 mothers with maternal justice involvement and 119 children of those women. The phases and processes for data linkages to establish the Maternal Justice Infant Data Hub are overviewed in Fig. 1.

## Discussion

The current case study presents the creation of novel cross-sector administrative data linkage that stands to overcome prior barriers in supporting rigorous inquiries of relationships between maternal justice involvement and child health. The linkage is an innovative approach to identify cohorts of children impacted by maternal incarceration to advance health justice and pediatric equity research to facilitate the study of these mechanisms as well as development of interventions to promote health and well-being within families impacted by maternal justice involvement. This case study is a ‘proof concept’ with limitations and barriers encountered. Lessons learned, and successes of this case study lend insights and a foundation for health and corrections data linkages to be replicated, expanded, and improved upon, to enhance research at the intersection of criminal justice and health.

These efforts are important, as the continuation of high incarceration rates of women represents a great threat to child health, as women still consistently bare the primary responsibility for parenting and caretaking for children in the US. Unsurprisingly, most women detained in state and federal prisons are parents (Maruschak, 2021). Even so, little is known about the effects of maternal incarceration on newborn and early childhood health (Herreros-Fraile et al., 2023; Wildeman et al., 2018), with mixed results on independent maternal incarceration effects (Poehlmann-Tynan & Turney, 2021; Wildeman & Turney, 2014). This can be partially explained by a lack of reliable data across outcomes and small sample sizes. Understanding the effects and mechanisms of maternal justice-involvement influencing early child health and well-being are critical to the development of effective public health responses, including evidence-based intervention and prevention strategies to mitigate risks and consequences for children exposed to maternal justice involvement.

State-wide administrative linkages (including health, correctional, child welfare, and family resource systems) similar to those in Wisconsin (Brown et al., 2011) would be an ideal strategy to overcome inherent and persistent problems with self-report data (currently the gold standard) about stigmatized and criminalized experiences that are subject to social desirability, under-reporting, and recall bias. However, this linkage would require substantial and sizeable investments to create and maintain. This level of investment is uncommon in many states. Within these more constrained contexts, efforts such as those presented in this case study represent cost-effective, replicable strategies to leverage existing, siloed data to support novel research and evaluation that can inform improved practices for optimizing and delivering healthcare services for families impacted by maternal or parental justice involvement. Within our case study, the cross-sector data linkage cost $45,000 in grant funding and took 1.5 years to complete both phases. As part of that $45,000, grant expenses covered two semesters of part time graduate students time, honest brokers to link and de-identify the cross-sector linkage data, community advisory board members time, and 5% of one year’s salary for the first and senior authors.

The cross-sector data linkage established within this case study is currently being used to lead several research inquiries designed to better understand the potential health consequences of maternal justice involvement on children as well as strategies to mitigate those impacts. Specifically, this data is currently being utilized to investigate health care use patterns, service connection, and common physical and mental health diagnoses among children of justice-involved mothers. Additionally, our study team seeks to identify and explore protective factors within these families that may be able to offset potential health consequences associated with maternal justice involvement. While not within the scope of the current study, similar methodologies could be utilized to establish cohorts that could be followed over time and would support innovative longitudinal study designs that would otherwise be impossible by leveraging traditional self-report data from one single sector (criminal justice, pediatric healthcare or adult healthcare). Importantly, efforts such as ours can also initiate greater mobilization beyond our county of interest to various state actors and administrators who may see more of the appeal.

The current case study should be considered within the context of some methodological limitations and lessons learned that researchers interested in creating health-justice data linkages within their own regions would be wise to consider. First, our direct efforts to obtain local county justice involvement records on women via the **masked for review** Clerk of Courts were not successful despite this information being publicly available. As a result, we were only able to obtain prior justice involvement data for women with active justice involvement via ODRC data. A successful partnership with the county’s clerk of courts could have allowed access to data on all female criminal justice records, which would have yielded a much larger linked sample. Scholars could invest time in building relationships and partnerships that can foster beneficial usage of these linked data through data management and storage support or guidance towards generating usable data. While this may be a good catalyst for establishing mutually beneficial partnerships in some cases, it may be met with resistance in other cases. Second, while we engaged a RAB early on to inform the ethical creation and use of this cross-sector data linkage prior to linkage; there is a need to formally investigate and interrogate the ethics (e.g. informed consent, beneficence, non-maleficence, and justice) of leveraging and linking publicly available correctional administrative data to healthcare administrative records without the consent of those who are incarcerated or justice-involved. Families affected by incarceration have been historically marginalized, harmed by prior unethical research, and excluded from participating in avenues of society and research. Despite our intentions to establish a linked dataset to create greater knowledge, inform positive health changes, and enhance support for families affected by incarceration and justice involvement, we express caution to researchers who leverage these approaches in their own city without inclusion of subject matter experts and/or communities within the city or state throughout the process. In these efforts, it is important to prioritize the potential unintended consequences through iterative review, and to treat the protection of families linked data as a critical, non-negotiable responsibility for all partners. With these considerations and challenges in mind, the creation of cross-sector datasets has the potential to generate local knowledge to inform better targeted health and social policies for families affected by incarceration within the county as well as enhance scientific rigor of research in this area.

## Conclusion

The creation of a novel cross-sector administrative data linkage that integrates correctional and health administrative records from corrections facilities, adult hospital systems, and pediatric hospital system proved feasible in this case study and offers a potentially replicable process to advancing research and policy aimed at improving child health and maternal outcomes within constrained funding landscapes. The ability to understand the intergenerational impacts of the carceral system are crucial to informing successful public health efforts to improve delivery and design of evidence-based interventions to promote child and family health when a family member becomes justice involved. As county, state, health and correctional systems increasingly recognize the importance of data-driven decision making and re-imagine and expand the evaluation of success beyond simply evaluating recidivism; the creation of cross sector data linkages represents a step toward more innovative and impactful research questions and responsive health and correctional policies. As the scientific field moves toward greater cross-sector data integration in other areas, especially beyond the county-level, continued efforts to refine linkage methodologies and ensure ethical creation and data governance will be essential in maximizing the potential of these datasets to drive meaningful social and health policymaking at the intersection of criminal justice and health.

## Funding and COI Statements

This work was supported by the National Center for Advancing Translational Sciences of the National Institutes of Health (2UL1TR001425). The content is solely the responsibility of the authors and does not necessarily represent the official views of the NIH. The author(s) declare(s) that they have no competing interests. This work is not under consideration for publication elsewhere, nor has it been previously published.

## Data Availability

Data is available via the existing Maternal Infant Data Hub and existing public records maintained by the respective study state and county; however, restrictions apply to the availability some these data, which were used under agreement for the current study and therefore, the resulting linked dataset is not available.
